# Adequate control of hypertension among older adults: ELSI-Brazil

**DOI:** 10.11606/S1518-8787.2018052000646

**Published:** 2018-10-25

**Authors:** Josélia Oliveira Araújo Firmo, Juliana Vaz de Melo Mambrini, Sérgio Viana Peixoto, Antônio Ignácio de Loyola, Paulo Roberto Borges de Souza, Fabíola Bof de Andrade, Maria Fernanda Lima-Costa

**Affiliations:** I Fundação Oswaldo Cruz. Instituto René Rachou. Núcleo de Estudos em Saúde Pública e Envelhecimento. Belo Horizonte, MG, Brasil; II Universidade Federal de Minas Gerais. Escola de Enfermagem. Belo Horizonte, MG, Brasil; III Fundação Oswaldo Cruz. Instituto de Comunicação e Informação Científica e Tecnológica em Saúde. Rio de Janeiro, RJ, Brasil; IV Fundação Oswaldo Cruz. Instituto René Rachou. Programa de Pós-Graduação em Saúde Coletiva. Belo Horizonte, MG, Brasil

**Keywords:** Aged, Hypertension, prevention & control, Socioeconomic Factors, Health Surveys, Idoso, Hipertensão, prevenção & controle, Fatores Socioeconômicos, Inquéritos Epidemiológicos

## Abstract

**OBJECTIVE:**

To estimate the prevalence of adequate control of hypertension among older adults and to examine its association with predisposing and enabling factors and the need to use health services.

**METHODS:**

The analysis was carried out with 4,148 participants (≥ 50 years) from the baseline of the Brazilian Longitudinal Study of Aging (ELSI-Brazil), who reported being hypertensive and using antihypertensive medication. Adequate control of hypertension was defined as systolic and diastolic blood pressure below 140 mmHg and 90 mmHg, respectively. The following exploratory variables were included: age, sex, health behaviors, and body mass index (predisposing factors); region of residence, rural or urban residence, education level, socioeconomic status of the household, and coverage by private health plan (enabling factors); and medical diagnosis of diabetes (need). The multivariate analysis was performed using Poisson regression and binary logistic regression.

**RESULTS:**

The prevalence of adequate control of hypertension was equal to 51.1% (95%CI 48.5–53.6). After adjusting for potential confounders, we observed statistically significant associations (p < 0.05) for education level > 4 years [prevalence ratio (PR) = 1.12 in relation to the lowest level], highest quintile of the socioeconomic status (PR = 1.22 in relation to the lowest quintile), coverage by private health plan (PR = 1.13), residence in the South (PR = 1.19) and Midwest regions (PR = 1.20) in relation to the Southeast region, and obesity (PR = 1.10).

**CONCLUSIONS:**

Half of the population studied had adequate control of hypertension. The improvement of this control is an important challenge, which should consider overcoming social and regional inequalities associated with it.

## RESUMO

**OBJETIVO:**

Estimar a prevalência do controle adequado da hipertensão arterial sistêmica entre adultos mais velhos e examinar a sua associação com fatores predisponentes, facilitadores e de necessidade para o uso de serviços de saúde.

**MÉTODOS:**

A análise foi conduzida em 4.148 participantes (≥ 50 anos) da linha de base do Estudo Longitudinal da Saúde dos Idosos Brasileiros (ELSI-Brasil), que informaram ser hipertensos e estar em uso de medicação anti-hipertensiva. O controle adequado da hipertensão arterial foi definido pela pressão sistólica e diastólica inferior a 140 mmHg e 90 mmHg, respectivamente. As variáveis exploratórias incluíram: idade, sexo, comportamentos em saúde e índice de massa corporal (fatores predisponentes); macrorregião de residência, residência rural ou urbana, escolaridade, situação socioeconômica do domicílio e cobertura por plano privado de saúde (fatores facilitadores); diagnóstico médico para diabetes (fator de necessidade). A análise multivariada foi feita por meio da regressão de Poisson e regressão logística binária.

**RESULTADOS:**

A prevalência do controle adequado da hipertensão arterial foi igual a 51,1% (IC95% 48,5–53,6). Após ajustes por potenciais variáveis de confusão, associações estatisticamente significativas (p < 0,05) foram observadas para escolaridade > 4 anos [razão de prevalência (RP) = 1,12 em relação ao nível inferior], quintil superior do nível socioeconômico (RP = 1,22 em relação ao quintil mais baixo), cobertura por plano privado de saúde (RP = 1,13), residência nas regiões Sul (RP = 1,19) e Centro-Oeste (RP = 1,20) em relação à região Sudeste, e obesidade (RP = 1,10).

**CONCLUSÕES:**

Metade da população estudada apresentou controle adequado da hipertensão arterial. A melhora desse controle é um importante desafio, que deve considerar a superação das desigualdades sociais e regionais a ele associadas.

## INTRODUCTION

Hypertension is the main risk factor for mortality and the third major cause of disability-adjusted life years (DALYs) in the world ^1^ . Despite the scientific and technological advances, its adequate control among treated persons remains an important challenge for public health^2–4^. This challenge arises from the complexity of the determinants of hypertension control, which include characteristics of the context, such as access to and use of health services and medication, and individual characteristics, such as adherence to prescribed medication and healthy lifestyles^5^ ^,^ ^6^.

The adequate control of hypertension is defined as systolic and diastolic blood pressure below 140 mmHg and 90 mmHg^2^ ^,^ ^3^ ^,^ ^7^, respectively, among those taking antihypertensive medication. In Canada and the United States, the prevalence of this control in adults (≥ 18 years) ranges from 67% to 69%^8^ ^,^ ^9^. In China, Mexico, South Africa, and Costa Rica, the corresponding prevalence for the age group of 50 years and over varies between 39% and 40%^2^. A similar prevalence (40%) was observed among older individuals (≥ 71 years old) in the Bambuí cohort (Brazil)^10^. A recent survey, using data from the Brazilian National Health Survey, has reported a lower prevalence (33%) for the Brazilian adult population (≥ 18 years)^5^. However, it is important to emphasize that this estimate was made considering all persons with hypertension, and not only those using antihypertensive medication^5^, as was the case in the above mentioned studies^2,8–10^.

The knowledge of the factors associated with the adequate control of hypertension allows the identification of vulnerable groups for secondary prevention. However, these factors seem to vary among populations. A study conducted in China showed that sociodemographic characteristics, health behaviors, and health insurance coverage were associated with adequate control of hypertension^11^. In the Bambui cohort study of aging, in Brazil, education level was associated with adequate control of hypertension among those aged ≥ 71 years, both in the cross-sectional analysis conducted in 1997 and in the one conducted in 2011^10^. A longitudinal analysis of the same cohort, comprising 11 years of follow-up of blood pressure among participants aged 60 years and over, has shown that family income was the only factor associated with the trajectory of adequate control of hypertension over other sociodemographic characteristics, health behaviors, presence of chronic diseases, and African and Native American genomic ancestry^12^. In the study with adult participants of the Brazilian National Health Survey, sex, education level, and other health conditions showed statistically significant associations with blood pressure < 140/90 mmHg among those who were aware they were hypertensive^5^. To our knowledge, no previous Brazilian study has investigated this topic in a nationally representative sample of older adults.

Our study aimed to estimate the prevalence of adequate control of hypertension among older Brazilian adults and predisposing factors, enabling factors, and the need to use health services associated with this control.

## METHODS

### Study Population

We used data from the baseline of the Brazilian Longitudinal Study of Aging (ELSI-Brazil), which is a national, home-based survey conducted in 2015-2016. The sampling design, which was designed to represent the Brazilian population aged 50 years and over, was based on stages, considering municipality, census tract, and household. For municipalities with up to 750,000 inhabitants, selection was carried out in three stages (municipality, census tract, and household). For larger municipalities, selection was carried out in two stages (census tract and household). The final sample consisted of 10,000 individuals (9,412 participants) living in 70 municipalities in different regions of the country. The participants of the ELSI’s baseline are similar to the Brazilian population aged 50 and over, in relation to age, sex, region of residence, and rural or urban residence, among other relevant characteristics^13^. For this analysis, we considered information obtained through interviews and physical measurements. More details on the methodology and descriptive results of the ELSI can be found on the research homepage^a^ and in another publication^13^.

### Study Participants

We selected all participants who reported having a medical diagnosis of hypertension and using antihypertensive medication. This information was obtained through the following questions: a) “Has any doctor told you that you have hypertension (high blood pressure)?”, and b) “Do you take medication for hypertension (high blood pressure)?”.

### Dependent Variable

The dependent variable of the study was adequate control of hypertension, defined by systolic pressure <140 mmHg and diastolic pressure < 90 mmHg^2^ ^,^ ^3^ ^,^ ^7^. To measure blood pressure, the participant remained seated and rested for at least five minutes without alcohol or caffeine intake for at least 30 minutes or exercise in the last hour. Three measurements were taken with two-minute intervals between them. The final measurement was considered as the mean of the second and third measurements.

### Covariates

The selection of covariates was based on the theoretical model of Andersen and Newman^14^, considering the predisposing factors [age, sex, health behaviors, and body mass index (BMI)], enabling factors (rural or urban residence, region of residence, education level, socioeconomic status, and private health plan coverage), need, and use of health services (medical diagnostic report of diabetes).

Health behaviors included current smoking, excessive alcohol consumption, regular consumption of fruits and vegetables, physical activity, and obesity, which are among the main risk factors for chronic non-communicable diseases, as contemplated in the national targets for the strategic action plan for coping with chronic non-communicable diseases in Brazil, 2011–2012^15^.

Those who reported smoking daily were considered as current smokers. Excessive alcohol consumption in the past 30 days was defined as the consumption of five or more doses of alcoholic beverages on a single occasion for men and four or more doses of alcoholic beverages on a single occasion for women. The intake of fruits and vegetables was defined by the answers to four questions, considering the number of days in the week and the number of daily portions consumed for each of these items. The recommended intake of these types of food was fixed as the intake of five or more servings on at least five days in the week^15^.

The level of physical activity was evaluated using the short version of the IPAQ (International Physical Activity Questionnaire) translated and validated for Brazil^16^. This questionnaire contains questions related to the frequency (days per week) and duration (time per day) of the physical activities performed in the week before the interview, including walking, moderate activities, and vigorous activities, considering only those performed for at least 10 continuous minutes at a time, including: (a) walking (at home or at work; as transport; for leisure, for pleasure, or as exercise), (b) moderate activities (light cycling; swimming; dancing; light aerobics; amateur volleyball; lifting light weights; gardening; etc., excluding walking), and (c) vigorous activities (running; aerobics; playing soccer; cycling fast; playing basketball; doing heavy chores in the house, yard, or garden; lifting weights; etc.). We converted these data into total time of practice of physical activity in the reported week, considering double the time spent in vigorous activities. Regular physical activity was defined as 150 minutes or more per week, as recommended by the World Health Organization^17^.

The BMI, which was obtained by dividing weight by squared height, was categorized according to recommendations of the Pan American Health Organization (WHO)^18^: low weight (BMI ≤ 23 kg/m^2^), normal weight (BMI > 23 kg/m^2^ and < 28 kg/m^2^), pre-obesity (BMI ≥ 28 kg/m^2^ and < 30 kg/m^2^), and obesity (BMI ≥ 30 kg/m^2^). Weight and height measurements were obtained in duplicate, and we considered the mean as the final product.

The area of residence was defined by the census tract of the household, which was adopted from the classification of the Brazilian Institute of Geography and Statistics (IBGE)^13^. Education level was categorized into less than four years and four years or more of study. Socioeconomic status was defined by a score of household goods, considering the number of equipment and vehicles in the household (refrigerator, washing machine, dishwasher, microwave oven, color television, VCR or DVD or similar, landline, cell phone, air conditioner, computer, cable or satellite TV, motorcycles and cars), in addition to the existence of domestic workers. This score was estimated through principal components analysis, in which higher values meant better conditions. For this analysis, we considered the distribution in quintiles. Health plan coverage was attributed to those who reported having private, company, or public agency health plan, except dental plan.

### Data Analysis

The association between independent variables and adequate control of hypertension was based on estimates of prevalence ratios (PR) and 95% confidence intervals (95%CI) estimated by robust Poisson regression. All described covariates simultaneously entered the final multivariate model, as they did not show evidence of collinearity (variance inflation factor < 5.0)^19^. Binary logistic regression was used to estimate the predicted probabilities of adequate control of hypertension by age, according to education level and socioeconomic status of the household.

All estimates were made considering the sample parameters and individual weights, using the *svy* procedure of Stata statistical package v.14.1.

### Ethical Aspects

The ELSI-Brazil was approved by the Research Ethics Committee of the *Instituto René Rachou* of the Oswaldo Cruz Foundation, Minas Gerais (CAAE 34649814.3.0000.5091). All individuals who agreed to participate in the study signed a specific informed consent form for each procedure carried out.

## RESULTS

Among the 9,412 participants of the ELSI’s baseline, 4,453 reported having a medical diagnosis of hypertension and using antihypertensive medication, and they were thus included in this analysis. Of them, 4,148 (93.2%) had complete information for all variables considered in this analysis and were included in the study. The prevalence of adequate control of hypertension was 51.1% (95%CI 48.5–53.6). The corresponding values for those aged 50-59, 60-69, and 70 years and over were 53.7% (95%CI 49.1–58.3), 51.7% (95%CI 48.8–54.6), and 46.9% (95%CI 43.5–50.3), respectively. The mean age of participants was 64.9 [standard deviation (SD) = 9.6], 60.2% were women, and 37.0% had less than four years of study. Other characteristics of the study participants can be seen in Table 1.

**Table 1:** Distribution of the characteristics of study participants. Brazilian Longitudinal Study of Aging (ELSI-Brazil), 2015–2016.

Variable	Total (%)	95%CI
Predisposing factors		

Age group in years		
50 to 59	37.0	33.5–40.6
60 to 69	34.1	32.3–36.0
70 and over	28.9	26.1–31.9
Female	60.2	57.5–62.9
Recommended level of physical activity^a^	66.7	63.9–69.5
Current smoker	12.2	10.8–13.6
Average weekly consumption of 5 or more servings of fruits and vegetables	16.9	15.1–18.9
Excessive alcohol consumption^b^	7.5	6.4–8.8
Body mass index (kg/m^2^)		
> 23 and < 28 (normal weight)	35.0	32.6–37.4
≤ 23 (low weight)	11.0	9.7–12.5
≥ 28 and < 30 (pre-obesity)	15.8	14.4–17.4
≥ 30 (obesity)	38.2	35.9–40.5

Enabling factors		

Urban area of residence	84.1	78.5–88.4
Region of residence		
Southeast	48.8	37.1–60.6
North	4.4	1.6–11.4
Northeast	23.2	15.2–33.7
South	16.6	8.8–29.1
Midwest	7.0	3.3–14.4
Education level in years		
< 4	37.0	33.4–40.7
≥ 4	63.0	59.3–66.6
Socioeconomic status (quintile)		
1st	19.5	16.0–23.6
2nd	21.1	19.2–23.2
3rd	20.4	18.2–22.8
4th	19.6	17.4–22.0
5th	19.4	16.7–22.5
Health insurance		
No	74.9	71.8–77.7
Yes	25.1	22.3–28.2

Need to use health services		

Medical diagnosis of diabetes		
No	78.5	76.5–80.4
Yes	21.5	19.6–23.5

^a^ ≥ 150 minutes/week.

^b^ Consumption in the last 30 days of five or more doses for men and four or more doses for women.

%: Percentages weighted by the sample parameters and weights of the individuals.

Number of respondents (unweighted): 4,148

Table 2 shows the results of the analyses of factors associated with adequate control of hypertension. In the unadjusted analysis, the following variables showed statistically significant associations (p < 0.05) with the outcome: age, education level, socioeconomic status of the household, region of residence, private health plan coverage, physical activity, and BMI. After mutual adjustments for all covariates considered in the study, we observed positive and statistically significant associations for education level of four years or more (PR = 1.12; 95%CI 1.03–1.22), socioeconomic status of the household (PR = 1.22; 95%CI 1.06–1.39 for those in the highest quintile), private health plan (PR = 1.13; 95%CI 1.04–1.22), residence in the South and Midwest regions in relation to the Southeast region (PR = 1.19; 95%CI 1.04–1.37 and PR = 1.20; 95%CI 1.01–1.41, respectively), and BMI equal to or greater than 30 kg/m^2^ (PR = 1.11; 95%CI 1.02–1.20).

**Table 2:** Association between adequate controla of blood pressure according to predisposing factors and enabling factors and the need to use health services. Brazilian Longitudinal Study of Aging (ELSI-Brazil), 2015–2016.

Variable	Adequate control of blood pressure				

	%	Crude PR^b^	95%CI	Adjusted PR^c^	95%CI
Predisposing factors					

Age group in years					
50–59	53.7	1.00		1.00	
60–69	51.7	0.96	0.87–1.06	0.99	0.89–1.09
≥ 70	46.9	0.87	0.80–0.96	0.91	0.83–1.01
Sex					
Female	52.0	1.00		1.00	
Male	49.7	0.96	0.88–1.05	0.94	0.86–1.03
Recommended level of physical activity					
No	47.8	1.00		1.00	
Yes	52.7	1.10	1.02–1.19	1.05	0.96–1.14
Current smoker					
Yes	49.6	1.00		1.00	
No	51.3	1.03	0.92–1.16	1.01	0.90–1.14
Weekly consumption of fruits and vegetables					
<5 servings daily, on average	50.8	1.00		1.00	
≥ 5 servings daily, on average	54.5	1.07	0.97–1.19	1.02	0.92–1.14
Excessive alcohol consumption					
Yes	50.9	1.00		1.00	
No	51.1	1.00	0.87–1.16	1.04	0.90–1.20
Body mass index (kg/m^2^)					
> 23 and < 28 (normal weight)	49.5	1.00		1.00	
≤ 23 (low weight)	48.1	0.97	0.87–1.09	1.02	0.89–1.15
≥ 28 and < 30 (pre-obesity)	48.6	0.98	0.86–1.12	0.98	0.86–1.10
≥ 30 (obesity)	54.4	1.10	1.01–1.19	1.11	1.02–1.20

Enabling factors					

Area of residence					
Urban	51.0	1.00		1.00	
Rural	51.3	1.00	0.92–1.10	1.05	0.95–1.17
Region of residence					
Southeast	48.5	1.00		1.00	
North	52.3	1.08	0.95–1.23	1.12	0.98–1.27
Northeast	49.7	1.03	0.92–1.14	1.12	0.99–1.26
South	57.7	1.19	1.05–1.35	1.19	1.04–1.37
Midwest	56.7	1.17	0.98–1.39	1.20	1.01–1.41
Education level in years (ref: < 4)					
< 4	45.0	1.00		1.00	
≥ 4	54.6	1.21	1.12–1.32	1.12	1.03–1.22
Socioeconomic status (in quintiles)					
1st quintile	46.2	1.00		1.00	
2nd quintile	47.8	1.03	0.92–1.16	1.00	0.89–1.12
3rd quintile	47.9	1.04	0.92–1.16	0.98	0.88–1.09
4th quintile	51.8	1.12	0.97–1.30	1.05	0.92–1.22
5th quintile	62.2	1.35	1.17–1.55	1.22	1.06–1.39
Health insurance					
No	48.8	1.00		1.00	
Yes	57.7	1.18	1.08–1.29	1.13	1.04–1.22

Need for the use of health services					

Medical diagnosis of diabetes					
No	51.7	1.00		1.00	
Yes	49.1	0.95	0.86–1.05	0.95	0.86–1.05

^a^ Systolic pressure <140 mmHg and diastolic pressure <90 mmHg between treated individuals.

^b^ PR (95%CI): crude prevalence ratio (95% confidence interval), estimated by the Poisson regression model.

^c^ PR (95%CI): adjusted prevalence ratio (95% confidence interval) for all variables listed in the table, estimated by the Poisson regression model.

%: Percentages estimated considering the sample parameters and the weights of the individuals.

**Figure 1:**
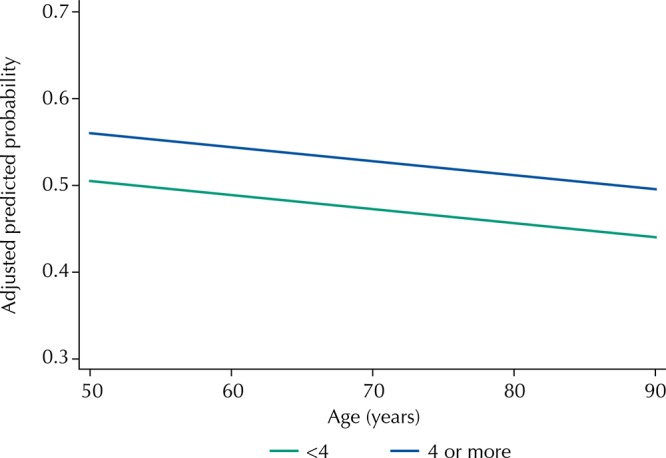
Predicted probability* of adequate control of hypertension by age and education level. Brazilian Longitudinal Study of Aging (ELSI-Brazil), 2015–2016.

**Figure 2:**
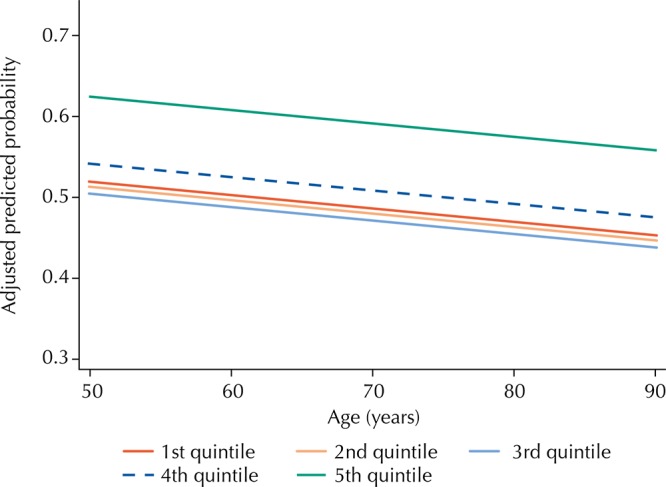
Predicted probability* of the adequate control of hypertension by age and socioeconomic status of the household. Brazilian Longitudinal Study of Aging (ELSI-Brazil), 2015–2016.

Figures 1 and 2 show the predicted probabilities of adequate control of hypertension by age, according to educational level and socioeconomic status of the household. We can observe that, at all ages, the predicted probability of adequate control of hypertension is higher for those with education level equal to or greater than four years. Regarding the socioeconomic status of the household, we observed a consistent better control at all ages among those in the highest quintile when compared to the lower quintiles.

* Estimated by binomial logistic regression and adjusted for all variables listed in Table 2.

* Estimated by binomial logistic regression and adjusted for all variables listed in Table 2.

## DISCUSSION

The results show that the prevalence of adequate control of hypertension among Brazilian older adults was lower than that observed among high-income countries^8^ ^,^ ^9^, but it was higher than that observed among other low- or middle-income countries^2^ ^,^ ^20^. Another relevant result refers to the important social inequalities observed in this control, in which higher prevalence was observed among those with higher education, living in households with better socioeconomic status, and with private health plan coverage. We also observed regional heterogeneity, in which the residents of the South and Midwest regions had better performance in relation to the Southeast region. These inequalities persisted after adjustment for all the variables included in the analysis.

As highlighted by Malta et al.^15^, the Brazilian Ministry of Health has implemented important policies to address non-communicable diseases (NCDs), particularly since 2006. In addition to the organization of the surveillance system, these measures included the National Policy for Health Promotion (published in 2006) focusing on healthy eating and physical activity, the Health Academy Program (created in 2011), and prevention of tobacco and alcohol use. At the same time, there was a significant expansion of the Family Health Strategy and pharmaceutical care with free distribution of medication for hypertension and diabetes, initially in the pharmacies of the basic units of the Brazilian Unified Health System (SUS) and later in private pharmacies (Popular Pharmacy Program)^15^. The Family Health Strategy is associated with greater access and satisfaction with the care and reduced hospitalizations and mortality for cerebrovascular disease and heart disease^21^. In Brazil, approximately seven out of 10 drugs used to treat hypertension were obtained from pharmacies of the SUS (56.0%) or the Popular Pharmacy Program (16.0%).^22^. Another important initiative is the Brazil without Misery program, whose objective is to reduce poverty, highlighting actions that address NCDs^15^.

The results of our analysis show that, despite these initiatives, the prevalence of adequate control of hypertension among older adults is relatively low (51%). On the other hand, when compared to other countries, our results show an intermediate position between high- (prevalence of 67–69%)^8^ ^,^ ^9^ and middle- or low-income counties (prevalence of approximately 40%)^2^ ^,^ ^20^. Thus, it is reasonable to suggest that the better Brazilian performance, in relation to the latter countries, is due to the aforementioned initiatives, particularly those related with care.

The World Health Organization (WHO) has drawn attention to the importance of social determinants of NCDs and their outcomes^15^. An analysis of 43 cohort studies has shown that – among six conventional risk factors – low socioeconomic status ranks third in relation to population attributable risks for mortality; smoking and physical inactivity rank first and second^23^. Social inequalities in health conditions of Brazilian older adults (60 years and over) are widely described^24^. Thus, it is not surprising that, in our analysis, education level and socioeconomic status of the household showed negative and independent associations with adequate control of hypertension with no differences by age. These iniquities can be explained by differences in income and education level in the determinants of adequate control of hypertension, which include complex factors such as access to and use of health services and medication, adherence to medical prescription, and adoption of healthy habits^5^ ^,^ ^6^.

Approximately 70% of the Brazilian older adults (≥ 60 years) use the SUS, but there are marked differences in the household income per capita among them^24^. In the highest income quintile, 29% exclusively use the SUS, while in the lowest quintile, the corresponding proportion increases to 92%^24^. A recent study has examined the hypertension care continuum among Brazilian adults (≥ 18 years) covered by private health plan, Family Health Strategy, and the traditional basic health unit. The care continuum is based on six indicators, which include the access, use, and quality of the services used. In general, the results showed a better performance of these indicators among those with private health insurance coverage and, for some of them, also among those under the Family Health Strategy^5^. However, we highlight that none of the three groups presented high hypertension quality care, which indicates that this care needs to be improved in Brazil ^5^. Our results are in line with these observations, since private health plan coverage was positively associated with better adequate control of hypertension.

There is evidence that the use of health services, access to medication, and adherence to educational programs for prevention vary across the Brazilian regions. The number of appointments performed by older adults is higher in the Southeast region and lower in the North and Midwest regions^25^. Full access to antihypertensive medications is high in urban areas of all regions (at least 96% among adults and 99% among older adults), but this access is even higher in the South region and lower in the Northeast and Midwest regions^22^. Partial access (only part of the prescribed medication) is higher in the Midwest (4%) and lower in the South (0.7%)^26^. Participation in educational activities offered under the Family Health Strategy also varies by region. Only 21.7% and 16.3% of the older adults with chronic diseases participate in these activities in the Northeast and Southeast regions^27^. Regional heterogeneities in the adequate control of hypertension observed in our analysis can be explained, at least in part, by these factors.

Although drug treatment has precise indications, the adoption of a healthy lifestyle is strongly suggested as a coadjuvant for the control of hypertension, with emphasis on the regular practice of physical activity, healthy eating, smoking cessation, and reduced alcohol consumption^28^ ^,^ ^29^. This indication is based on evidence that these behaviors may reduce blood pressure levels, improve the effect of antihypertensive medication, and decrease cardiovascular risk, and this effect is intensified when two or more practices are combined^30^_._ A study with data from the VIGITEL survey in Brazil (Surveillance of Risk Factors and Protection for Chronic Diseases by Telephone Survey) has shown that health behaviors did not differ between older adults aware of their hypertension and those who reported not having the disease^31^. The only exception was smoking, whose prevalence was lower among the former than the latter^31^. Among the health behaviors considered in our analysis, only physical activity was associated with adequate control of hypertension, but the association disappeared after adjustment for potential confounding factors.

The literature shows that BMI is a risk factor for hypertension^4^ and a study carried out in China has found a positive association between adequate control of hypertension and BMI^3^. The results of this analysis show a paradox, that is, obese older adults (BMI ≥ 30 kg/m^2^) have a better control of hypertension. One hypothesis to explain the association is that obese hypertensive persons are more likely to seek health services or use antihypertensive medication as prescribed by the physician.

Among the limitations of this study, as well as those who have examined the adequate control of hypertension in large population-based studies^10^ ^,^ ^11^, we can mention the self-reported nature of the information on the treatment of hypertension. Thus, we cannot rule out the possibility of classification bias, which may reduce the strengths of the observed associations. The literature shows that adherence to drug treatment is strongly associated with adequate control of hypertension^32^_._ In our study, we could not examine this adherence, which represents another limitation. Another limitation is its cross-sectional nature, which does not allow temporal relations to be established. However, it is important to point out that the association between socioeconomic status and adequate control of hypertension, observed in this analysis, is in line with that observed in a longitudinal analysis of the Bambuí cohort study of aging ^10^. The main advantage of our study is its population base, which is representative of the non-institutionalized Brazilian population in the eligible age group, thus allowing for the first time in the country the examination of the prevalence and some factors associated with the adequate control of hypertension in the population aged 50 years and over.

In summary, the adequate control of hypertension among Brazilian older adults remains a major challenge, since only half of the treated population shows blood pressure levels at recommended levels. Maintenance of the free supply of antihypertensive medication is very important so that progress can be made to control hypertension, but additional actions are needed to ensure adherence to the prescribed medication, the continuous care of treated persons, and the promotion of healthy habits. Finally, it is important to highlight that our results support the view that improvements in education level and income can substantially contribute to improve the control of hypertension.

## Footnotes

b Fundação Oswaldo Cruz. Brazilian Longitudinal Study of Aging. Rio de Janeiro; c2015 [cited 2017 Nov 28]. Available from: http://elsi.cpqrr.fiocruz.br
